# The dual‐role clinician teacher: A human bridge for socially responsible AI use

**DOI:** 10.1111/medu.70035

**Published:** 2025-09-09

**Authors:** Hiske Joanna Brouwer, Margot Barry, Esther de Groot

**Affiliations:** ^1^ Department of General Practice & Nursing Science, Julius Center for Health Sciences and Primary Care, University Medical Center Utrecht Utrecht University Utrecht The Netherlands

## Abstract

Underscoring the importance of social learning in health professions education, the authors suggest ways for clinician teachers to take advantage of their unique position to model and embed social learning practices in AI‐integrated education.

Clinician teachers operate at a unique intersection: They are deeply embedded in both clinical practice and medical education. In both ‘worlds’, the use of Generative AI (GenAI) has entered and transformed, if not revolutionised, daily work. In this commentary, we centre on the clinician teacher's role in mediating and connecting GenAI's influence and use in both worlds. More specifically, after briefly outlining current applications of GenAI in medical education and clinical practice, we argue for the vital contribution of the clinical teacher in fostering socially grounded, critically engaged AI use.

The systematic review published in this issue^1^ sheds a welcome light on how health professional education students make use of GenAI and how this aligns with Laurillard's Conversational Framework. GenAI tools are being rapidly adopted for finding and exploring information, deepening understanding of complex concepts, generating materials and seeking feedback. As Pham et al. showed, the tools used mostly support individualised learning, enabling flexible and self‐directed study. However, the study's findings suggest a decrease in collaborative, dialogic learning and social interactions, as discussion and collaboration were a lot less frequently reported. The shift towards more individualised learning risks reinforcing a model of medical learning centred on solitary interaction with digital agents—an ironic regression to a time when the literature on health professions education felt the need to advocate for more teamwork, situated learning and shared reflection.^2^

The shift towards more individualised learning risks reinforcing a model of medical learning centred on solitary interaction with digital agents.

Simultaneously, as GenAI is being used by students in education, it is also gaining ground in the clinical domain. Yim et al.^3^ conducted a systematic narrative review on the use of GenAI in health care and found multiple applications. GenAI can assist clinicians in disease detection using a variety of imaging techniques, in generating summaries of patient encounters, in translating medical jargon into patient‐friendly explanations to support communication during visits and in summarising the medical history of patients. Also, GenAI can guide physicians, for instance, by suggesting diagnostic or treatment steps, or proposing possible diagnoses based on symptoms and patient history. Lastly, the authors found GenAI could automatise certain processes, such as transcribing and summarising clinical conversations. Here too, GenAI largely serves the individual professional rather than the team. Based on this review, we concluded that GenAI is typically used in isolation, outside of multidisciplinary deliberation and often without feedback loops or peer critique. In doing so, GenAI tools add to the risk of reinforcing an image of the clinician as a solo performer—at odds with the collaborative, team‐based reality of contemporary health care.

GenAI tools add to the risk of reinforcing an image of the clinician as a solo performer—at odds with the collaborative, team‐based reality of contemporary health care.

1

The growing individualised use of GenAI in both education and clinical practice presents a challenge for health professions education. As Bleakley et al. argued nearly two decades ago, medical education must evolve beyond transmission‐based learning towards social, collaborative learning^2^; a vision fortunately considered obvious in our time, even though embedding this in the composition of our medical curricula remains difficult. However, GenAI's current use risks pulling learners back into personal cognitive pathways, diminishing social interaction, critical thinking and learning to cope with the uncertainty inherent to patient care.

GenAI's current use risks pulling learners back into personal cognitive pathways, diminishing social interaction and critical thinking.

Here, the clinician teacher, as dual‐role professional, has a unique opportunity to intervene. Our realist review^4^ on the merits of the dual role demonstrated that learning occurs during social processes such as during dialogue and reflection, role modelling and learning in socio‐cultural contexts. This underscores the value of a clinical teacher, not just in the transfer of knowledge, but by co‐creating knowledge through interaction, relationships and real‐world engagement.

Clinician teachers have first‐hand experience in observing how GenAI is being integrated into both patient care and student learning. Their dual perspective grants them both the insight and the responsibility to guide students in using AI critically, collaboratively and professionally. While it is unrealistic to expect clinician teachers to be experts in GenAI—especially given the demands of their dual roles—they are well positioned to facilitate and participate in meaningful dialogue with students, who often feel quite comfortable with these technologies. These conversations can explore how GenAI influences clinical reasoning and information trust in ways that foster *mutual* learning.

These conversations can explore how GenAI influences clinical reasoning and information trust in ways that foster *mutual* learning.

How might a clinician teacher intervene? Here, we outline two opportunities for an active role. First, while we know GenAI offers powerful support, it is also known to occasionally produce outdated or incorrect information and can exhibit hallucinations in certain contexts.^5^ And, as Holm^6^ notes, AI‐generated outputs for individual patients are based on large datasets and often fail to incorporate contextual factors or patient values. A growing concern is that students may adopt its—often confidence‐inducing and well‐formulated—output uncritically, undermining the development of their critical thinking skills. To mitigate this, clinical teachers should allocate time to help students become aware of such inaccuracies, which are sometimes easily recognisable, but certainly not always. Clinician teachers might design assignments that require students to engage with GenAI, compare its output to traditional academic sources and peer feedback and facilitate conversation about the differences. Not only do these tasks create opportunities to correct inaccuracies and foster deeper critical thinking skills, but it also enables them to reflect collectively on epistemic uncertainties: When is an answer ‘good enough’, what counts as valid knowledge and are inaccuracies a phenomenon of the AI area only? When clinician teachers are open with the students about their own epistemological challenges while using GenAI, they create rich opportunities for bidirectional learning.^7^

Second, GenAI diminishes the chance of experiencing uncertainty. GenAI tools always produce a clear response to your query; however, in patient care and clinical reasoning, answers are often not binary or immediately clear. As a result, students have fewer opportunities to develop uncertainty tolerance, a critical skill in diagnostic reasoning and professional resilience.^8^ Clinician teachers can create activities that provide dialogue around uncertainty. For instance, students could bring in complex clinical cases and use GenAI to generate several diagnostic or therapeutic options. Rather than focusing solely on the truthfulness of generated options, clinician teachers might facilitate a discussion around how much certainty is required before acting. Does this threshold shift depending on clinical context, and where do GenAI's suggestions align (or fail to align) with human reasoning?

Since GenAI tools are here to stay in both medical education and patient care, we propose that clinicaian teachers have a critical integrative role to play in shaping GenAI's future in health professions education. They are connectors—a human bridge—who understand how learning conversations about GenAI are indispensable in a critical approach towards learning and patient care. Their unique vantage point, knowledgeable in both contexts where AI is being adopted, makes them ideal candidates to model and embed social learning practices in AI‐integrated education: co‐constructing understanding, questioning sources and emphasising social learning.

Clinician teachers, knowledgable in both contexts where AI is being adopted, makes them ideal candidates to model and embed social learning practices in AI‐integrated education.

## AUTHOR CONTRIBUTIONS

**Hiske Joanna Brouwer:** Conceptualization; writing—original draft. **Margot Barry:** Conceptualization; supervision; writing—review and editing. **Esther de Groot:** Conceptualization; supervision; writing—review and editing.
